# High-dose versus low-dose ergocalciferol for correcting hypovitaminosis D after fragility hip fracture: a randomized controlled trial

**DOI:** 10.1186/s12877-021-02023-1

**Published:** 2021-01-21

**Authors:** Atthakorn Jarusriwanna, Suchat Phusunti, Pojchong Chotiyarnwong, Aasis Unnanuntana

**Affiliations:** 1 Department of Orthopaedics, Faculty of Medicine, Naresuan University, 99 Moo 9, Phitsanulok-Nakhon Sawan Road, Mueang Phitsanulok, Phitsanulok, 65000 Thailand; 2 Chok Chai Hospital, 220 Moo 13, Omsin Road, Chok Chai, Nakhon Ratchasima, 30190 Thailand; 3 Department of Orthopaedic Surgery, Faculty of Medicine Siriraj Hospital, Mahidol University, 2 Wang Lang Road, Bangkok Noi, Bangkok, 10700 Thailand

**Keywords:** Ergocalciferol, Hypovitaminosis D, 25-hydroxyvitamin D, 25(OH)D, Fragility hip fracture

## Abstract

**Background:**

Hypovitaminosis D can be observed in most fragility hip fracture patients. However, measurement of 25-hydroxyvitamin D (25(OH)D) level is costly and may not be available in some centers. Without the baseline serum 25(OH)D level, the appropriate dose of vitamin D supplementation is not known. The aim of this study was to evaluate the effectiveness and safety of vitamin D supplementation in fragility hip fracture patients compared between high- and low-dose vitamin D supplementation.

**Methods:**

A total of 140 patients diagnosed with fragility hip fracture were randomly allocated to either the high-dose (60,000 IU/week) or low-dose (20,000 IU/week) vitamin D2 supplementation group for 12 weeks. The number of patients who achieved optimal vitamin D level (serum 25(OH)D > 30 ng/mL), the proportion of patients who developed hypercalcemia, and the functional outcome were compared between groups.

**Results:**

Of the 140 patients who were enrolled, 21 patients were lost to follow-up during the study period. The remaining 119 patients (58 and 61 in the high- and low-dose groups, respectively) were included in the final analysis. The high-dose group had a higher rate of serum 25(OH)D restoration to optimal level than the low-dose group (82.8% vs 52.5%, respectively; *p* < 0.001). Approximately 3.4 and 1.6% of patients in the high- and low-dose groups, respectively, had mild transient hypercalcemia, but none developed moderate, severe, or symptomatic hypercalcemia. There were no differences in functional outcome scores between groups.

**Conclusions:**

In treatment settings where baseline serum 25(OH)D level can’t be evaluated in older adults with fragility hip fracture, we recommend high-dose vitamin D2 of approximately 60,000 IU/week for 12 weeks, with subsequent switch to a maintenance dose. This regimen effectively restored serum vitamin D to an optimal level in 82.8% of patients without causing symptomatic hypercalcemia.

**Trial registration:**

The protocol of this study was retrospectively registered in the Thai Clinical Trials Registry database no. TCTR20180302007 on 20 February 2018.

## Background

Vitamin D is a fat-soluble vitamin that plays a major role in musculoskeletal health [1]. The main function of vitamin D is to regulate calcium and phosphate balance by enhancing intestinal absorption and renal reabsorption, to maintain bone strength, and to modulate muscle function via receptors that are expressed in muscle cells [2]. Previous studies found hypovitaminosis D to be associated with several musculoskeletal-related problems, especially postural instability, falling among the older adults, and osteoporosis [3]. Prolonged vitamin D deficiency causes secretion of parathyroid hormone, leading to secondary hyperparathyroidism, which is one of the common causes of secondary osteoporosis.

Vitamin D deficiency is a common condition worldwide with a prevalence ranging from 41.6 to 87.4%, and older adults with fragility hip fracture are among those at greatest risk [4, 5]. Previous literature demonstrated that 78.4% of patients with fragility hip fracture had hypovitaminosis D [6], and a meta-analysis study showed that patients with a low serum 25-hydroxyvitamin D (25(OH)D) level had significantly increased risk of hip fracture [7]. With a global projection of 2.6 million hip fractures occurred annually by the year 2025 [8], the number of patients with insufficient vitamin D would increase exponentially. It is, therefore, crucial to correct low vitamin D status in all patients with osteoporotic hip fracture.

In general, vitamin D supplementation is available in 2 forms: vitamin D2 (ergocalciferol) and vitamin D3 (cholecalciferol) [9]. Although these 2 formulas are different in their side chain structure, both forms are effective in restoring serum vitamin D level [10]. The reason that we used vitamin D2 in this study is because vitamin D2 is the main formula available in Thailand. When treating hypovitaminosis D, measurement of serum 25(OH)D is required to determine the appropriate dose of vitamin D supplementation. However, this laboratory test is not available in many centers. Moreover, the cost to measure serum 25(OH)D level is much higher than the cost of vitamin D supplementation itself. Therefore, some physicians preliminarily administer vitamin D without determination of serum 25(OH)D level before treatment. The effectiveness and safety of this strategy, however, is not known.

The objective of this study was to compare the effectiveness and safety between different doses (high- and low-dose) of vitamin D2 supplementation in fragility hip fracture patients. The clinical relevancy of this study is that if high-dose vitamin D supplementation is safe and more effective to restore optimal vitamin D status in older adults with fragility hip fracture, physicians can pretreat all patients with high-dose vitamin D without frequent determination of serum 25(OH)D level.

## Methods

The study protocol and consent forms were approved by the Institutional Review Board (IRB), and registered in the Thai Clinical Trials Registry (registration no. TCTR20180302007). The study design and reporting format were based on Consolidated Standards of Reporting Trials (CONSORT) guidelines. Patients aged older than 50 years who were diagnosed with pertrochanteric hip fracture (either intertrochanteric or femoral neck fracture) during October 2016 to November 2017 were screened for the study. The exclusion criteria were patients with hypercalcemia (corrected serum calcium more than 10.5 mg/dL), renal impairment with estimated glomerular filtration rate (eGFR) less than 30 mL/min/1.73 m^2^, abnormal liver function test with aspartate aminotransferase (AST) or alanine aminotransferase (ALT) level greater than twice the upper limit of the normal range, previous vitamin D supplementation, and pathological fracture, defined as fracture in patients who had a history of any bone tumor either primary or metastasis. This condition was suspected in patients with osteolytic/osteoblastic lesion around the hip from preoperative radiographs. In addition, specimens from the fracture site were sent for pathological examination in most cases to confirm osteoporotic fracture. Patients who met all of the eligibility criteria were randomized using a computer-generated system with block sizes of four to receive vitamin D (ergocalciferol 20,000 IU capsule, British Dispensary, Bangkok, Thailand) either high- or low-dose for 12 weeks.

The demographic data included gender, age, body mass index (BMI), type of hip fracture (intertrochanteric fracture or femoral neck fracture), treatment option either conservative or surgical procedures according to fracture type. The characteristics of previous functional status and activity (outdoor or indoor), and pre-operative ambulatory status whether patient could ambulate most time of the day independently without gait aid, depending on gait aids (single cane, tripod cane, quad cane, or pick up walker), or unable to walk were asked.

Patients in the low-dose vitamin D group received vitamin D2 20,000 IU (1 capsule) per week, while high-dose vitamin D group patients received 60,000 IU (3 capsules) per week. Generally, vitamin D was administered after surgical intervention or when the surgeon chose to treat a hip fracture conservatively. In addition, all patients received calcium carbonate 1000 mg per day. Baseline serum 25(OH)D, total calcium, albumin, phosphorus, and parathyroid hormone (PTH) levels were measured prior to supplementation and at 12 weeks after the first dose of vitamin D. After the completion of this study, all patients in both treatment groups were switched to a maintenance dose of vitamin D2 supplementation (20,000 IU per week). The research assistants who collected data were blinded to each patient’s supplementation protocol.

### Outcome measurement

The primary outcome of this study was the proportion of patients who achieved optimal vitamin D level at 12 weeks after vitamin D supplementation. The serum 25(OH)D concentration level was classified as hypovitaminosis D if the serum 25(OH)D level was < 30 ng/mL [11]. The quality assurance protocol of laboratory assays in this study was under the Thailand National External Quality Assessment Scheme (NEQAS) in clinical chemistry standard, which collaborated with the World Health Organization International External Quality Assessment Scheme (WHO IEQAS) [12, 13]. Serum 25(OH)D and PTH were measured and analyzed by electrochemiluminescence (ECL) binding assay on a cobas 8000 analyzer (Roche Diagnostics GmbH, Mannheim, Germany). The intra-assay coefficient of variation (CV) of serum 25(OH)D measurement at 10.6 and 29.0 ng/mL were 8.5 and 3.3%, respectively, while the inter-assay CV at 10.6 and 29.0 ng/mL were 9.2 and 4.5%, respectively. For measurement of serum PTH level, the intra-assay CV at 49.3 and 160 pg/mL were 0.9 and 1.5%, respectively, while the inter-assay CV at 49.3 and 160 pg/mL were 0.8 and 1.3%, respectively. Total serum calcium was analyzed by 5-nitro-5′-methyl-BAPTA assay on a cobas 8000 analyzer (Roche Diagnostics GmbH, Mannheim, Germany), which the intra-assay CV at 9.3 and 14.6 mg/dL were 0.7 and 0.5%, respectively, while the inter-assay CV at 9.3 and 14.6 mg/dL were 0.8 and 0.9%, respectively. Hypercalcemia was classified as mild, moderate, or severe based on corrected serum calcium, which was calculated based on serum albumin level by adding 0.8 mg/dL of total serum calcium for every 1 mg decrease in serum albumin below 4 mg/dL, as described in the formula: corrected serum calcium (mg/dL) = measured total serum calcium (mg/dL) + [4.0 – serum albumin (g/dL) × 0.8] [14]. Mild hypercalcemia was defined as corrected serum calcium between 10.5 and 12 mg/dL, moderate hypercalcemia was defined as corrected serum calcium from 12 to 14 mg/dL, and severe hypercalcemia was clarified when corrected serum calcium > 14 mg/dL [15]. Symptomatic hypercalcemia was diagnosed when corrected serum calcium was > 10.5 mg/dL with one of the following symptoms/signs: neurological dysfunction, myopathy, bradyarrhythmia, or gastrointestinal problems [16]. Functional outcome was evaluated using the Barthel Index and the EuroQol-visual analogue scale (EQ-VAS).

The Barthel Index is a measurement tool that is used to assess activities of daily living (ADLs). It comprises of 10 variables describing ADLs and mobility. Each item is rated by a scoring range which depended on performance of the patient. A higher score (maximum 100) indicates good functional ability and a greater likelihood of being able to live independently at home after discharge from the hospital, and a lower score (minimum 0) indicates poor functional status and likelihood of being dependent on caregiver. This tool has been validated in hip fracture patients [17].

EQ-VAS is a simple self-evaluated scale that rates health status ranging from 0 to 100 points. Patients rated their health status on a visual analogue scale, with a higher score (maximum 100) indicating the best imaginable health state, and a lower score (minimum 0) indicating the worst health status [18]. Similar to the Barthel Index, EQ-VAS was reported to be a reliable tool and has been validated in hip fracture patients [19].

### Statistical analysis and sample size calculation

Statistical power was considered from the primary outcome, which was the proportion of patients with adequate level of serum 25(OH)D after supplementation for 12 weeks. A study by Sansanayudh N et al. [20] showed that among patients with metabolic syndrome who supplemented with vitamin D2 20,000 IU per week for 8 weeks, 33.3% were restored to optimal vitamin D level, whereas 60% of patients who received vitamin D2 40,000 IU per week for 8 weeks were able to achieve optimal vitamin D level. Based on the results of that study, power analysis and sample size calculation indicated that a sample size of 54 patients per group would provide 80% statistical power (a one-sided alpha = 0.05; beta = 0.2). Recruitment was increased by 30% to compensate for a high incidence of loss to follow-up and death in this patient population. Therefore, a total of 140 patients were required for randomization in this study.

Baseline characteristics and all outcome measures are presented as number and percentage for categorial variables, and as mean ± standard deviation (SD) for continuous variables. The normality of data was assessed with Kolmogorov-Smirnov test. Pearson’s chi-square test or Fisher’s exact test was used for comparison of categorial variables, while Student’s *t*-test was used to compare continuous variables. Statistical analysis was performed using SPSS® Statistics version 18.0 (SPSS Inc., Chicago, IL, USA), and statistical significance was defined at a *p*-value of ≤ 0.05.

## Results

**Fig. 1:**
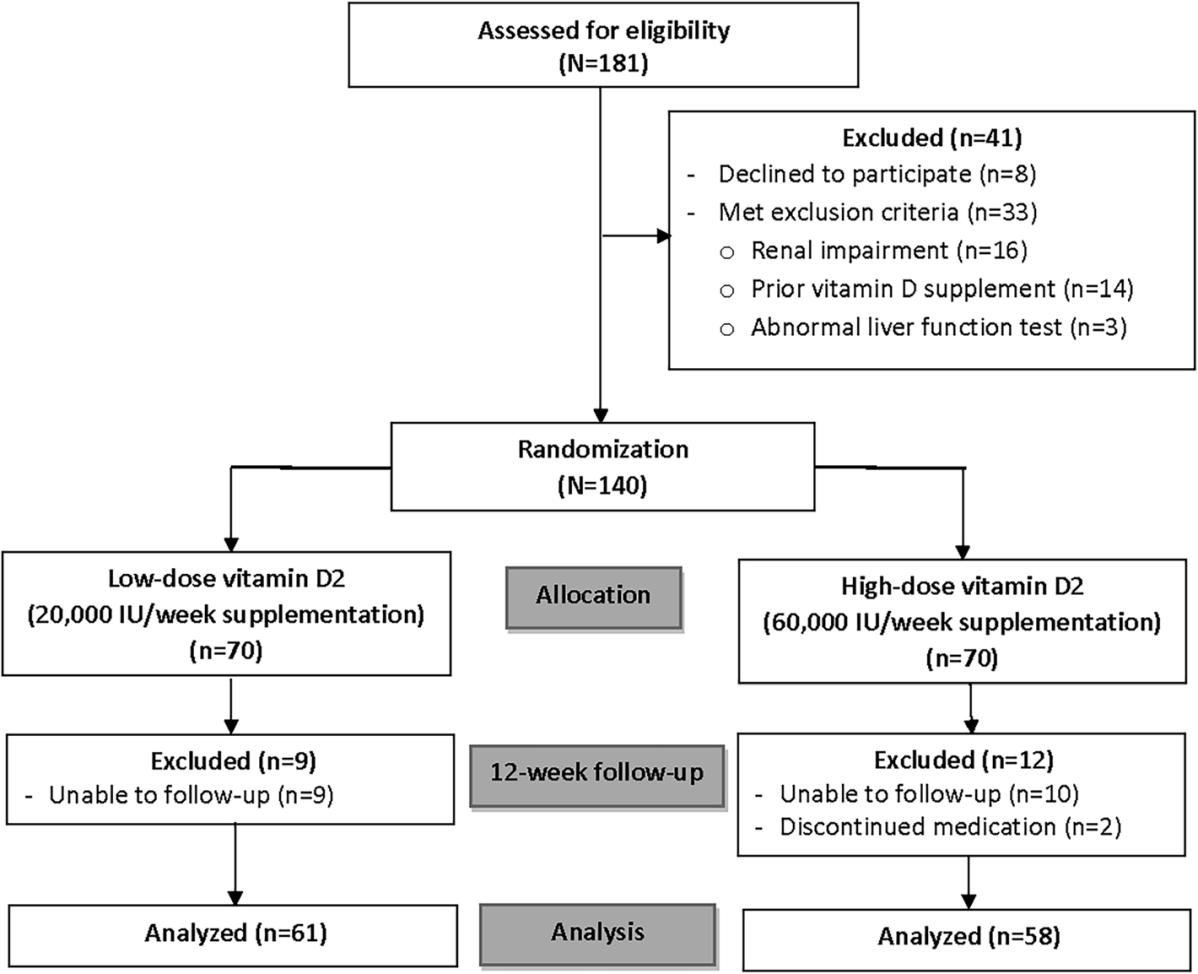
Consolidated Standards of Reporting Trials (CONSORT) diagram showing the flow of patients in the study

From 181 eligible patients, 41 were excluded for either not wanting to participate (*n* = 8) or for meeting one of the exclusion criteria as follows: 16 patients for renal impairment, 14 patients for receiving vitamin D supplementation prior to the study, and 3 patients for abnormal liver function test. From 140 randomized patients, 9 and 10 patients in the low- and high-dose vitamin D groups, respectively, were unable to come for follow-up, and 2 patients in the high-dose group discontinued their medication. Therefore, 119 patients (85%) completed the study with data available for analysis at the end of the 12-week study period (Fig. 1). Demographic and baseline characteristics of patients in both groups are shown in Table 1. The overall mean age of the patients was 78.9 ± 10.3 years, and most patients (72.1%) were female. Just over half (51.4%) of patients were diagnosed with intertrochanteric femoral fracture, and 48.6% had femoral neck fracture. Most patients were treated operatively, while 2 patients in the high-dose group and 1 patient in the low-dose group were treated conservatively. There were no significant differences in demographic and clinical characteristics between groups (Table 1).

**Table 1:** Patients demographic and clinical characteristics

Clinical variables	Low-dose group (*n* = 70)	High-dose group (n = 70)	*p-*value
Female gender, n (%)	52 (74.3)	49 (70.0)	0.575
Age (years), mean ± SD	78.5 ± 9.3	80.1 ± 10.0	0.311
Body mass index (kg/m^2^), mean ± SD	22.7 ± 3.6	22.5 ± 4.2	0.731
Charlson comorbidity index (CCI)			
- 0–1	46 (65.7)	47 (67.1)	0.840
- 2–3	23 (32.9)	21 (30.0)	
- > 3	1 (1.4)	2 (2.9)	
Type of hip fracture, n (%)			
- Intertrochanteric fracture	34 (48.6)	38 (54.3)	0.112
- Femoral neck fracture	36 (51.4)	32 (45.7)	
Treatment, n (%)			
- Conservative	1 (1.4)	2 (2.9)	0.691
- Multiple screw fixation	3 (4.3)	2 (2.9)	
- Dynamic hip screw fixation	8 (11.4)	4 (5.7)	
- Intramedullary nailing	30 (42.9)	33 (47.1)	
- Arthroplasty	28 (40.0)	29 (41.4)	
Previous functional status, n (%)			
- Outdoor	48 (68.6)	40 (57.1)	0.235
- Indoor	22 (31.4)	30 (42.9)	
Pre-operative ambulatory status, n (%)			
- Independent without gait aid	43 (61.4)	39 (55.7)	0.372
- Single cane	16 (22.9)	14 (20.0)	
- Tripod cane	3 (4.3)	6 (8.6)	
- Quad cane	0 (0)	0 (0)	
- Pick up walker	7 (10.0)	8 (11.4)	
- Wheel chair	0 (0.0)	2 (2.9)	
- Bed bound	1 (1.4)	1 (1.4)	

**Fig. 2:**
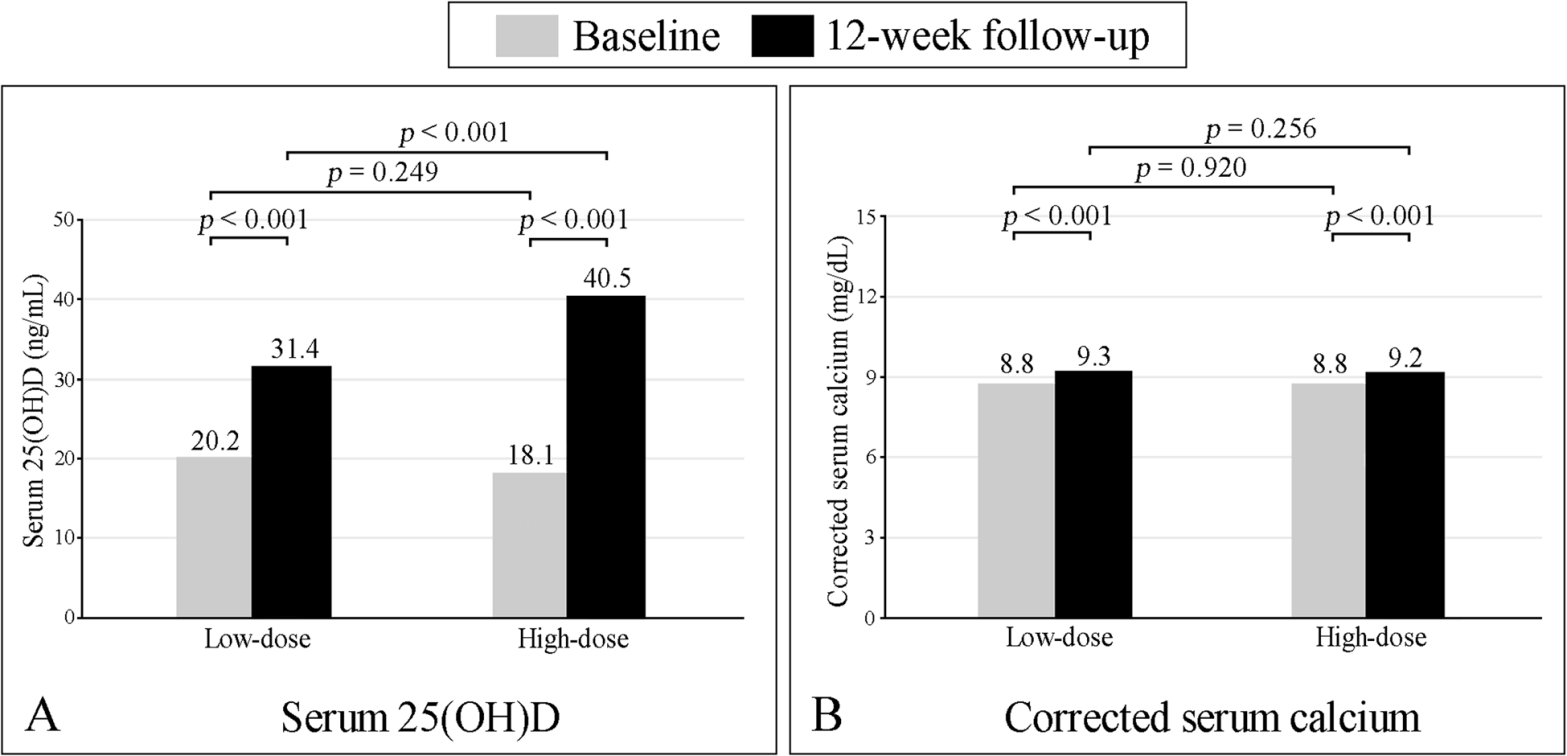
Graphs showing mean serum 25(OH)D (**a**) and mean corrected serum calcium (**b**) levels at baseline and at the 12-week follow-up

**Fig. 3:**
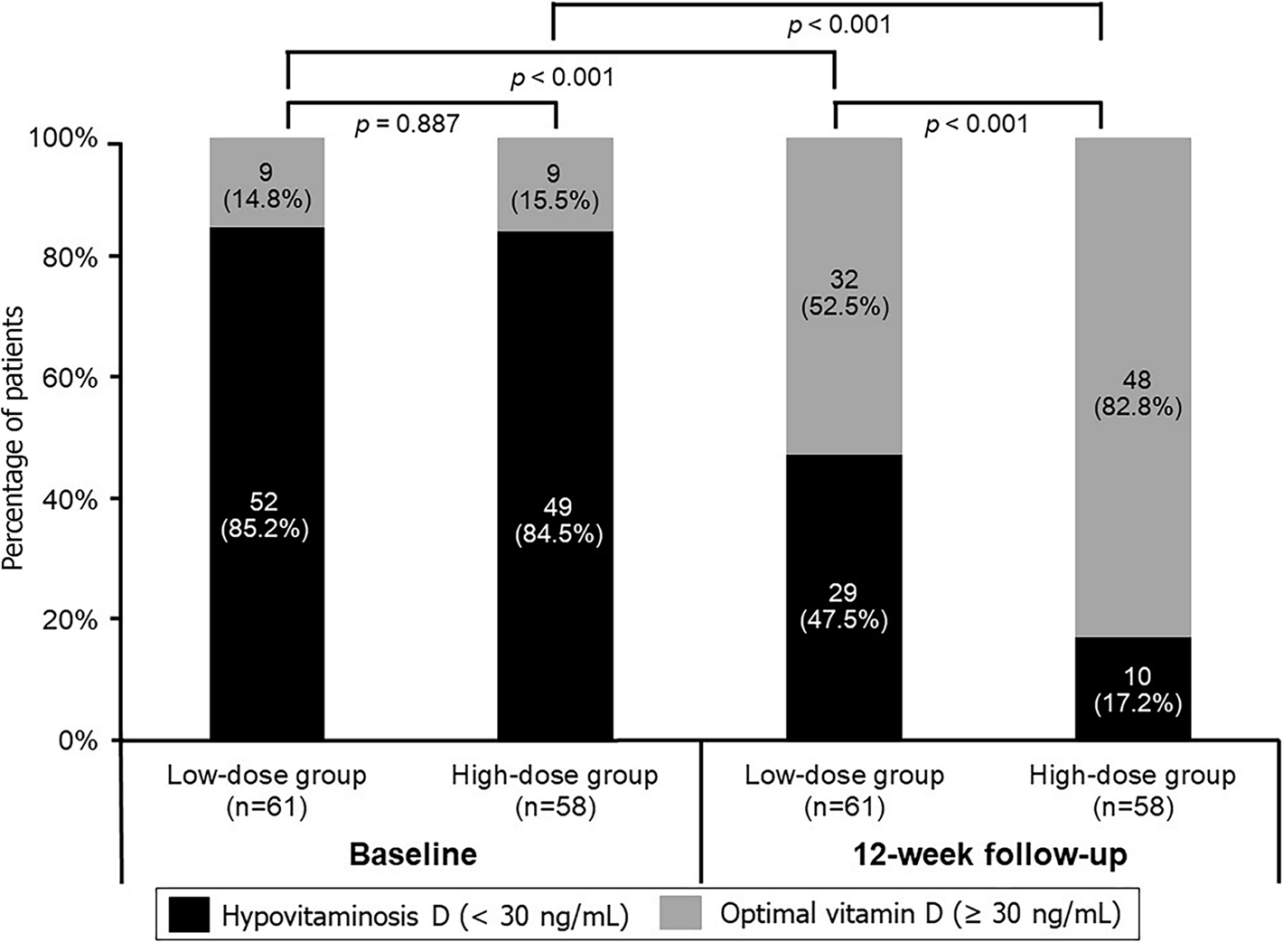
Proportion of patients with hypovitaminosis D and optimal vitamin D levels at baseline and at the 12-week follow-up

Baseline mean serum 25(OH)D levels in the low- and high-dose groups were 20.2 ± 8.2 ng/mL and 18.1 ± 11.1 ng/mL, respectively (*p* = 0.249). At the completion of the study, the mean serum 25(OH)D levels significantly increased to 31.4 ± 8.8 and 40.5 ± 12.5 ng/mL in the low- and high-dose groups, respectively. There was a significant difference in the post-treatment level of serum 25(OH)D between the 2 groups (*p* < 0.001) (Fig. 2a). The number of patients who achieved optimal serum 25(OH)D level was greater in the high-dose group significantly (82.8 and 52.5% for the high- and low-dose groups, respectively; *p* < 0.001) (Fig. 3).

The baseline mean serum PTH levels were 41.3 ± 18.4 pg/mL and 50.8 ± 25.3 pg/mL in the low- and high-dose groups, respectively. At the 12-week follow-up, the average PTH levels remained within the normal range in both groups (51.8 ± 36.0 pg/mL in the low-dose group, and 56.6 ± 55.5 pg/mL in the high-dose group). There were no significant differences in serum PTH levels between groups, or when compared between before and after vitamin D supplementation. Mean corrected serum calcium levels increased significantly from baseline to 12 weeks in both groups (8.8 ± 0.4 mg/dL to 9.3 ± 0.5 mg/dL in the low-dose group, *p* < 0.001; and 8.8 ± 0.6 mg/dL to 9.2 ± 0.6 mg/dL in the high-dose group, *p* < 0.001) (Fig. 2b). Two patients in the high-dose group and 1 patient in the low-dose group developed mild hypercalcemia (10.5 to 11.1 mg/dL), which was transient and asymptomatic (Table 2).

**Table 2:** Characteristics of patients who developed mild asymptomatic hypercalcemia after vitamin D supplementation

Patient no.	Group allocation	Baseline laboratory value				12-week follow-up laboratory value			
		Serum 25(OH)D (ng/mL)	eGFR (mL/min/1.73 m^2^)	Corrected serum calcium (mg/dL)	Serum phosphorus (mg/dL)	Serum 25(OH)D (ng/mL)	eGFR (mL/min/1.73 m^2^)	Corrected serum calcium (mg/dL)	Serum phosphorus (mg/dL)
1	Low-dose	20.7	84.6	9.8	3.1	29.4	82.2	10.5	3.4
2	High-dose	17.4	42.6	10.1	3.5	45.0	43.3	11.1	3.7
3	High-dose	8.1	85.4	10.0	2.9	34.2	82.7	10.8	3.3

**Fig. 4:**
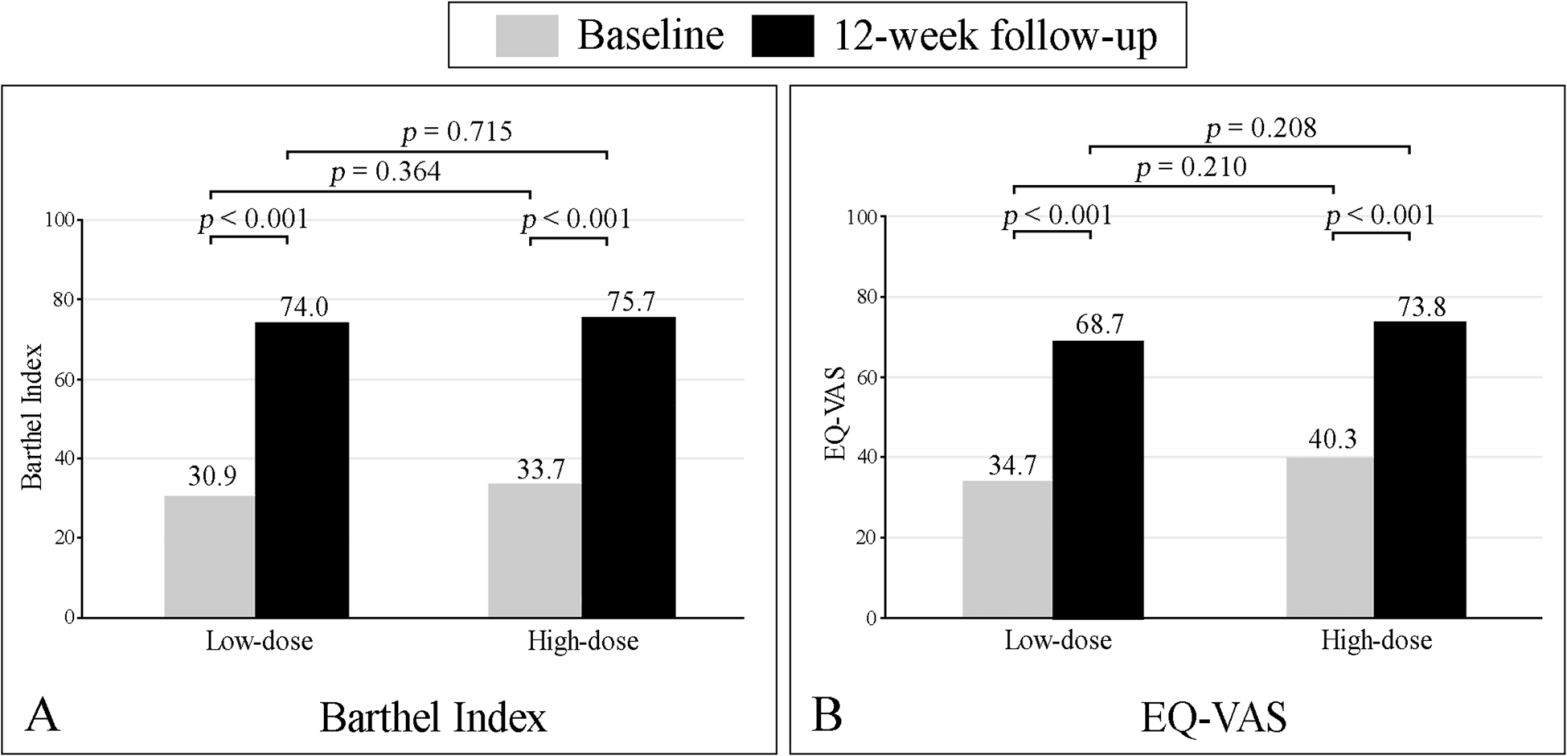
Graphs showing mean Barthel Index (**a**) and mean EuroQol-visual analog scale (EQ-VAS) (**b**) at baseline and at the 12-week follow-up

Regarding functional outcome scores, both Barthel Index and EQ-VAS were significantly improved from baseline to 12-weeks post-treatment in both groups. However, there was no significant difference when comparing between the 2 groups (Fig. 4a, b). The ambulatory status at 12-week after supplementation was also no significant difference when comparing between the 2 groups (Table 3).

**Table 3:** Functional and ambulatory status of patients after 12-week follow-up

Clinical variables	Low-dose group (*n* = 61)	High-dose group (*n* = 58)	*p-*value
Functional status, n (%)			
- Bed bound	6 (9.8)	4 (6.9)	0.374
- Outdoor	41 (67.2)	35 (60.3)	
- Indoor	14 (23.0)	19 (32.8)	
Ambulatory status, n (%)			
- Independent without gait aid	28 (45.9)	21 (36.2)	0.514
- Single cane	5 (8.2)	5 (8.6)	
- Tripod cane	0 (0.0)	2 (3.4)	
- Quad cane	0 (0.0)	0 (0.0)	
- Pick up walker	20 (32.8)	22 (37.9)	
- Wheel chair	2 (3.3)	4 (6.9)	
- Bed bound	6 (9.8)	4 (6.9)	

## Discussion

This study compared the effectiveness and safety between high-dose (60,000 IU per week) and low-dose (20,000 IU per week) vitamin D2 supplementation in fragility hip fracture patients for a 12-week duration and found that more proportion of patients in the high-dose group achieved optimal serum 25(OH)D level without complication of moderate to severe or symptomatic hypercalcemia.

Treatment of hypovitaminosis D is essential for osteoporosis patients, especially those with history of fragility fractures. A study by LeBoff MS et al. [21] showed that vitamin D deficiency was associated with low muscle strength, especially in the lower extremities, which affected patient stability and increased risk of falls. Treatment of hypovitaminosis D alone could reduce the fall rate by 14% [22]. Moreover, a meta-analysis study by Yao P et al. [23] demonstrated that an increased serum 25(OH)D level of 10 ng/mL was related to 20% lower risk of hip fracture. Given that older adults with fragility hip fracture had a high incidence of hypovitaminosis D (ranging from 78.4 to 92%) [6, 24], it is, therefore, important to treat this condition in order to prevent future complication.

The effectiveness of different doses of vitamin D supplementation has been evaluated by previous studies and benefit of high-dose vitamin D supplementation was shown. For instance, Mak JC et al. [25] retrospectively reviewed 124 patients with fragility hip fracture who had vitamin D deficiency and found that high-dose vitamin D3 supplementation (approximately 4000 IU per day for 14 days) improved serum 25(OH)D level to an optimal status in 88.9% of patients; however, only 62.5% of patients who received vitamin D3 1000 IU per day achieved optimal vitamin D level. A randomized controlled trial that compared the different doses of vitamin D3 also found that a high-dose vitamin D3 of 2000 IU per day for 12 months was able to restore serum 25(OH)D level to an optimal level in 93% of subjects, while only 70% of those who received a low-dose of vitamin D3 (800 IU per day) reached an optimal vitamin D status [26]. Although, the efficacy of vitamin D2 and vitamin D3 in maintaining serum 25(OH)D was comparable [10], there is limited data available specific to the effectiveness of vitamin D2 supplementation in different doses, especially in fragility hip fracture patients. Woranitat W et al. [27] reported a significant difference in the percentages of postmenopausal women with hypovitaminosis D who achieved optimal vitamin D level after 12 weeks of supplementation. More than three-quarters (86.4%) of patients who received vitamin D2 supplementation of 40,000 IU per week could reach optimal vitamin D level, whereas 44.0 and 27.3% of patients who took vitamin D2 of 20,000 IU per week and per 2 weeks, respectively, could achieve optimal vitamin D level. Another study by Sansanayudh N et al. [20] also reported superior results of high-dose vitamin D2 supplementation of 40,000 IU per week for 8 weeks in metabolic syndrome patients with vitamin D deficiency. Similar to those previous reports, our results showed that more patients in the high-dose vitamin D group achieved optimal vitamin D status (82.8 and 52.5% for the high- and low-dose groups, respectively).

Regarding the potential risk of high-dose vitamin D supplementation, alteration in serum calcium can lead to hypercalcemia. Previous study reported that patients with a serum 25(OH)D level exceeding 150 ng/mL were at risk for developing vitamin D intoxication or severe hypercalcemia [28]. Importantly, none of the patients in our study had serum 25(OH)D above 70 ng/mL. Only 2 patients in the high-dose group and 1 patient in the low-dose group developed mild hypercalcemia, which was transient and asymptomatic in all 3 cases. Thus, further investigation for the cause of hypercalcemia was not conducted.

Another interesting point is the level of serum PTH after vitamin D supplementation. The results from our study showed no suppression of serum PTH in according to increase level of serum 25(OH)D. This was probably because the effect of fracture healing process. Ban ZN et al. [29] demonstrated that hip fracture patients in a healing effective group had a significantly higher serum PTH level than delayed healing patients.

Therefore, our high-dose vitamin D supplementation protocol is safe and effective for the treatment of hypovitaminosis D in fragility hip fracture patients. In settings where there is limited access for serum vitamin D measurement, physicians can preliminarily prescribe a high-dose vitamin D2 (60,000 IU per week) for 12 weeks and then switch to a maintenance dose of vitamin D2 (20,000 IU per week). It is important to emphasize that a maintenance dose of vitamin D is required after a period of high-dose vitamin D supplementation to maintain optimal vitamin D status [11]. Bacon CJ et al. [30] reported the long-term sustainability of serum 25(OH)D level via a maintenance dose of vitamin D3 of 50,000 IU per month following a single bolus dose of vitamin D3 500,000 IU.

There is a trend toward increased serum vitamin D measurement worldwide [31]. This increased number of laboratory tests substantially impact healthcare expenditures [32]. In Australia, the cost of vitamin D measurement increased an average of 59% each year, with a forecasted total cost of vitamin D measurement of A$95.6 million in 2010 [33]. In the United States, there was an 83-fold increase in the reimbursement volume for serum 25(OH)D tests from 2000 to 2010 [34]. Similarly – in the UK, the measurement of serum vitamin D was increased approximately 50-fold from 2005 to 2015 [35]. Since the laboratory cost of vitamin D measurement is quite high, the protocol to check serum vitamin D, both pre- and post-treatment, has burdened many medical centers. At our center, the cost of each serum 25(OH)D test is approximately US$30. With an estimated 42,000 hip fractures in Thailand in 2018 [36], evaluating serum vitamin D before and after supplementation would cost approximately US$2,520,000 per year. Since our preliminary high-dose vitamin D supplementation is effective and safe, this protocol is a cost-effective strategy for treating low vitamin D status in older adults with fragility hip fracture without frequent measuring serum vitamin D level.

There were some limitations in this study. First, several confounders, such as dietary vitamin D intake and sunlight exposure, were not evaluated or controlled. However, patients with advanced age, lack of physical activity, various comorbidities, and frailty are likely to reduce their exposure to sunlight [37]. We, therefore, assumed that sunlight exposure not to be a major factor for changes in serum 25(OH)D level in this patient population. Second, there was a high drop-out rate in this study as expected, which is common among geriatric patients. Nevertheless, there were no differences in baseline patient demographics and clinical characteristics between patients who withdrew and those who completed the study. Third, there are a number of techniques to measure serum 25(OH)D level, and high performance liquid chromatography (HPLC) is considered a standard method [38]. However, the ECL technique used in our study showed good agreement with HPLC, and has been widely used in many centers [39]. Another limitation of serum 25(OH)D evaluation is that the laboratory measurement generally represented total 25(OH)D including both vitamin D2 and D3. Interpretation of serum 25(OH)D level should consider carefully, which negative bias may be observed and related to the concentration of vitamin D2 [40]. Fourth, the follow-up duration in this study was only 12 weeks in duration, so it is possible that a longer-term analysis may have shown a change in serum vitamin D level after switching patients to a maintenance dose. Last, the average baseline serum 25(OH)D level in this study was not extremely low (baseline mean serum 25(OH)D around 18–20 ng/mL); therefore, our high-dose vitamin D supplementation protocol might achieve lower vitamin D status if patients have an initial serum 25(OH)D level less than that of our study population.

## Conclusions

Vitamin D supplementation in patients with fragility hip fracture is essential. In settings where measurement of serum 25(OH)D is not available, a short period of high-dose vitamin D supplementation is a safe and effective protocol in older adults with fragility hip fracture. Based on the results of this study, we recommend treating all fragility hip fracture patients with ergocalciferol 60,000 IU per week for 12 weeks, and then switching them to a maintenance dose of 20,000 IU per week.

## Data Availability

The datasets used and/or analyzed during the current study are available from the corresponding author on reasonable request.

## Abbreviations

25(OH)D: 25-hydroxyvitamin D
IU: international unit
CONSORT: consolidated standards of reporting trials
eGFR: estimated glomerular filtration rate
AST: aspartate aminotransferase
ALT: alanine aminotransferase
BMI: body mass index
NEQAS: national external quality assessment scheme
WHO IEQAS: World Health Organization international external quality assessment scheme
PTH: parathyroid hormone
ECL: electrochemiluminescence
CV: coefficient of variation
EQ-VAS: EuroQol-visual analogue scale
ADLs: activities of daily living
SD: standard deviation
HPLC: high performance liquid chromatography
